# Facilitators and Barriers of Accessibility and Utilization of Healthcare Facilities in India: A Qualitative Metasynthesis

**DOI:** 10.7759/cureus.92183

**Published:** 2025-09-12

**Authors:** Aviraj KS, Manvi Sagar, Sarit Sharma, Debkumar Pal

**Affiliations:** 1 Community Medicine, Saraswathi Institute of Medical Sciences, Hapur, IND; 2 Community Medicine, Maharishi Markandeshwar College of Medical Science &amp; Research (MMCMSR) Sadopur, Ambala, IND; 3 Community Medicine, Dayanand Medical College and Hospital, Ludhiana, IND; 4 Community Medicine and Family Medicine, All India Institute of Medical Sciences, Kalyani, Kalyani, IND

**Keywords:** healthcare accessibility, healthcare system, healthcare utilization, network analysis, qualitative meta-analysis

## Abstract

India's healthcare system, a blend of public and private providers, faces deep-rooted inequities that hinder access for many. Rural areas lack adequate resources, while financial constraints prevent countless families from affording private care. This qualitative metasynthesis was conducted to explore and analyze the facilitators and barriers that shape access and utilization to both government-run and private healthcare establishments in India, delving deep into the insights provided by qualitative research methodologies.

In March 2024, we systematically searched PubMed, Scopus, Embase, and Google Scholar databases for qualitative studies related to facilitators and barriers of accessing government or private healthcare facilities in India. We included qualitative studies and mixed-method studies that reported themes and subthemes related to facilitators and barriers toward accessing government or private healthcare facilities in India. Methodological quality and the risk of bias were assessed using the Critical Appraisal Skills Programme (CASP) checklist for qualitative studies, followed by a comprehensive thematic analysis to distill and synthesize the findings gleaned from the studies. We finally included 36 articles fulfilling the inclusion criteria from 3771 identified records. Our review identified 10 overarching themes, each related to various facilitators and barriers to healthcare access and utilization in India. Facilitators included care accessibility, facility cleanliness, effective information sharing, and staff behavior, while barriers encompassed systemic challenges, patient factors, and physician-related issues. The findings suggest the presence of significant obstacles that impede efficient healthcare delivery and patient satisfaction. Our study emphasized the need for holistic, system-wide interventions that recognize the dynamic nature of healthcare access and can lead to lasting improvements for India's diverse population. Future research should assess the impact of government initiatives and explore strategies to ensure equitable healthcare for India’s diverse population.

## Introduction and background

Access to quality healthcare is a fundamental right, encompassing timely access to personalized services for optimal health outcomes [1]. With the adoption of the 2030 Sustainable Development Goals (SDGs), achieving Universal Health Coverage (UHC) became a global target, aiming for comprehensive healthcare access regardless of financial constraints [2].

India's healthcare reality is marked by a complex interplay of public and private sectors, collectively serving the diverse needs of the nation [3]. Government-run facilities aim to provide affordable care, while the private sector offers a spectrum of services, albeit often at varying costs [4]. Primary health centers, community health centers, and district hospitals are predominantly managed by the government, whereas the private sector, constituting 62% of India's health infrastructure, spans from high-end facilities to community clinics [5].

Despite strides, challenges persist, especially in rural areas where infrastructure, personnel, and medicine supply pose hurdles. Moreover, while the private sector fosters innovation, financial barriers limit access for many [6].

Significant differences in availability, accessibility, and utilization are revealed by India's healthcare system, which has a complex triple structure comprising government, private, and voluntary health agencies [7]. Overcrowding, outdated infrastructure, and staff shortages that erode patient trust are common problems in the state-run public sector, which is frequently beset by management deficiencies, manpower shortages, and a lack of accountability [7].

On the other hand, the private sector, which is favored by about 70% of the population, is mainly unregulated, leading to high fees and significant out-of-pocket (OOP) costs [8]. Interestingly, there are almost twice as many private hospitals in India as there are public ones. The glaring urban-rural divide makes these problems worse. About 80% of healthcare professionals and roughly 75% of health infrastructure are concentrated in urban areas, which is why the majority of people living in rural areas are underserved [9]. They are forced to travel long and difficult distances. India does not meet its own government standards as it has 16% fewer primary health centers and roughly 50% fewer community health centers than required. These draw attention to important gaps in the accessibility and availability of high-quality healthcare [9].

Understanding factors influencing healthcare-seeking behaviors is crucial for crafting responsive systems. This research delves into facilitators and barriers to accessing and utilizing government or private healthcare in India, aiming to inform policy formulation and service enhancement. By synthesizing insights from qualitative studies, it seeks to unravel complexities beyond statistical trends and foster a healthcare landscape that caters to India's diverse needs. We aimed to explore the facilitators and barriers to accessing and utilizing government or private healthcare in India from the available literature using a metasynthesis approach.

## Review

Methodology

Study Design

We conducted this metasynthesis to generate evidence on facilitators and barriers in accessing and utilizing government or private healthcare services in India. We prospectively registered the protocol for this review with the International Prospective Register of Systematic Reviews (PROSPERO; Identifier: CRD42024530024).

Databases and Search Strategy

We developed database-specific search strategies for identifying records from PubMed, Scopus, Embase, and Google Scholar (Appendices). We used keywords such as "facilitators," "utilization," "barriers," "India," and appropriate Boolean operators. The "PECOS" (Population, Exposure, Comparator, and Outcome) structure was used to formulate a research question: "What are the primary facilitators and barriers of accessing government or private healthcare facilities in India, as identified through qualitative studies?”

Inclusion and Exclusion Criteria

*Inclusion criteria: *We included qualitative studies or mixed-method studies that reported facilitators and/or barriers related to accessibility and/or utilization of government and/or private healthcare facilities in India. We included studies published in the period of 2013-2023 in the English language.

*Exclusion criteria: *We excluded studies with only a quantitative analysis, studies conducted outside India, and studies that reported the utilization and/or accessibility of a specific type of healthcare service, such as noncommunicable disease, human immunodeficiency virus (HIV), and anemia.

Identification and Selection of Studies

We rigorously identified and removed duplicate articles from our included studies manually. Titles and abstracts were screened for relevance by two independent reviewers. Full-text reviews were then conducted by two independent reviewers. Adjudication was done by one of the senior researchers in case of conflicts.

Risk of Bias Assessment

We used the Critical Appraisal Skills Programme (CASP) Qualitative Checklist to assess the quality of the included studies regarding potential bias (Appendices). Two independent reviewers assessed quality of each of the studies. Any disagreements were resolved through adjudication by another reviewer [10].

Data Extraction

Two researchers independently extracted relevant data using a predesigned Microsoft Excel sheet (Microsoft Corp., Redmond, WA). We extracted the themes, subthemes, and codes related to barriers and facilitators from the included studies. Two reviewers independently extracted the data using a structured data extraction format, followed by verification by another reviewer.

Data Cleaning and Integrity Check

We took a really close look at our dataset to make sure it was complete and accurate. We did not find any missing information, which means the data is high-quality and reliable. This thorough check ensures we can use it for our detailed analysis without needing to fill in any gaps or make corrections.

Data Analysis

Content analysis was conducted using R software (R Foundation for Statistical Computing, Vienna, Austria), identifying the common themes reported in the included studies. Text mining techniques were used to scrutinize word frequencies and co-occurrences. We identified quotes related to the common themes. We generated word clouds based on themes, subthemes, and codes related to barriers and facilitators using NVivo software (Lumivero, Burlington, Massachusetts). The interrelationship between facilitators and barriers influencing healthcare access and utilization was depicted using a network graph, constructed using R software, applying the tidyverse, dplyr, igraph, and ggraph packages. Codes related to facilitators and barriers alongside weights representing the relative prominence or frequency of each thematic linkage were processed to create two essential components: a nodes dataset, containing unique facilitator and barrier codes with attributes identifying their type, and an edges dataset, defining the thematic relationships and their weights.

A Fruchterman-Reingold force-directed layout was applied to the graph to minimize node overlap, ensuring clear clustering of related themes. Nodes were color-coded, with facilitators rendered in soft green and barriers in soft red, while edge thickness and opacity were scaled according to the strength of association. A Sankey diagram was generated to illustrate the directional flow between facilitators and barriers identified in the metasynthesis. Using networkD3 and htmlwidgets in R, facilitators were aligned on the left side of the visualization (depicted in blue), while barriers were aligned on the right side (depicted in red). Links, or ribbons, connected facilitators to barriers, with link width proportional to the weight of their thematic connection. While the network graph shows relationships and clustering, the Sankey diagram emphasizes directionality of thematic influence by establishing a directional flow, visualizing prominence, and identifying recurrent facilitator and barrier interactions.

Results

Search Strategy and Study Selection

**Figure 1:**
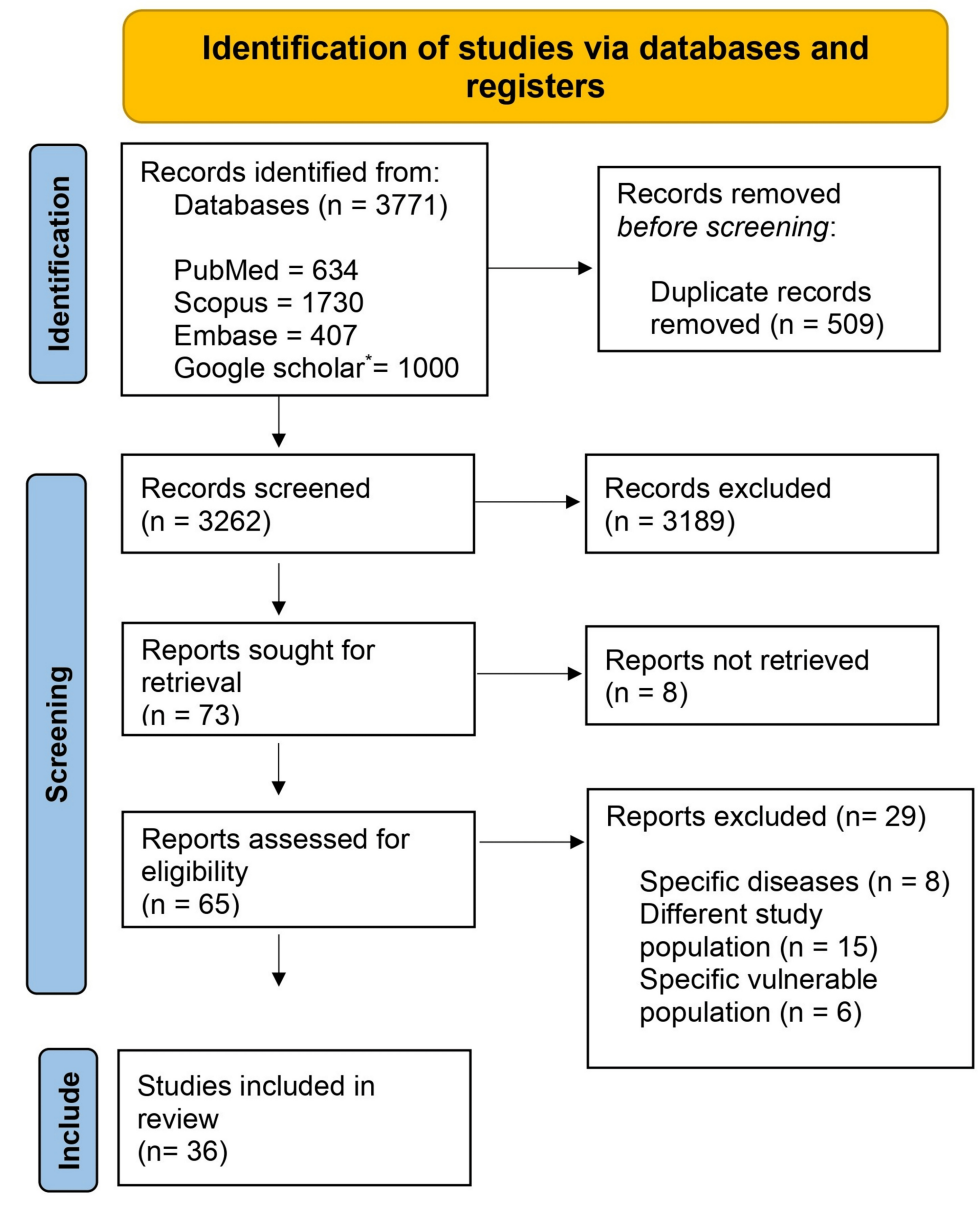
PRISMA flow diagram showing the study selection procedure * Only the first 1000 articles were selected.

A comprehensive electronic database search across PubMed, Embase, Google Scholar, and Scopus initially yielded 3771 articles. Manual searches were conducted on reference lists of pertinent articles and systematic reviews to enhance the search strategy. After removing duplicates and screening titles/abstracts, 73 articles were selected for full-text review. Of these, 36 qualitative studies met eligibility criteria for inclusion in the systematic review. The PRISMA flowchart illustrates the study selection process (Figure 1).

Baseline Characteristics of Included Studies

Each of the included studies utilized qualitative research methodologies to examine the facilitators and barriers related to accessing and utilizing healthcare facilities in India. Qualitative methods included interviews [11-39], focus group discussions [13-15,26,34,35,37,40-42], surveys [43], semistructured questionnaires [43-45], and participant observations [11-13,22]. Various sampling techniques were employed in this systematic review to capture diverse perspectives and experiences of individuals accessing healthcare services. Sampling methods ranged from purposive sampling [11,17,19,20,22,25,28,29,30-38,42,43,45], where participants were selected based on specific criteria relevant to the research question, to snowball sampling [18,24,46], where participants were recruited through referrals from initial participants. Additionally, convenience sampling was utilized, selecting participants based on their accessibility and willingness to participate. Participants encompassed a broad spectrum of demographics, including individuals from urban and rural settings, diverse socioeconomic backgrounds, different age groups, and various ethnic or cultural identities. The included studies spanned diverse regions in India, reflecting differences in healthcare infrastructure, government and private facilities, cultural norms, and socioeconomic factors (Table 1).

**Table 1:** Basic characteristics of the included studies (n = 36)

S. No.	Author (First author)	Year of publication	Study area	Study design	Study population	Sample size	Tool for data collection	Type of sampling	Type of analysis
									(Thematic/content)
1	Bhattacharyya et al. [38]	2015	Uttar Pradesh, India	Qualitative study	Recently delivered women and healthcare providers	40	In-depth interviews	Purposive sampling	Thematic analysis
2	Varghese et al. [46]	2015	SadholiKadim block, rural Uttar Pradesh, India	Qualitative study	Caregivers of children with intellectual disabilities	10	Semistructured interviews	Convenience and snowball sampling	Thematic analysis
3	Gawde et al. [44]	2016	Mumbai, India	Mixed-method study	Migrant women who had delivered in the last two years	234	Structured interview schedules, qualitative in-depth interviews	Multistage cluster sampling	Thematic/content analysis
4	Vidler et al. [45]	2016	Karnataka, India	Qualitative study	Women of reproductive age, community health workers, and health system representatives	46	Focus group discussions and one-to-one interviews	Purposive sampling	Thematic analysis
5	Merugumala et al. [21]	2017	Hyderabad, India	Qualitative study	Parents and clinic staff related to the care of hearing-impaired children	25	Semistructured interviews	Opportunistic sampling	Thematic analysis
6	Nielsen et al. [22]	2017	Tamil Nadu, India	Qualitative study	Pregnant women	39	Semistructured interviews and observations at health facilities	Purposive sampling	Content analysis
7	Vellakkal et al. [43]	2017	Jharkhand, Madhya Pradesh, Uttar Pradesh (India)	Qualitative study	Eligible women, their spouses, mothers-in-law, and ASHAs (Accredited Social Health Activists)	112	In-depth qualitative interviews	Purposive sampling	Thematic analysis
8	Rath et al. [27]	2018	India	Qualitative study	Oral cancer patients	70	Face-to-face in-depth interviews using a semistructured questionnaire	Convenience sampling	Thematic analysis
9	Patel et al. [31]	2018	Bihar, India	Qualitative study	Scheduled Caste (SC) women	18	Semistructured interviews	Purposive sampling	Thematic/content using framework analysis
10	Siddaiah et al. [36]	2018	Haryana, India	Mixed-method study	Migrant women laborers working in brick kilns	500	Surveys/questionnaires	Purposive sampling	Thematic/content analysis
11	Elias et al. [40]	2018	Tumkur, Karnataka, India	Mixed-methods study	Households and healthcare workers	1149	Household and health facility surveys, focus group discussions (FGDs), and in-depth interviews	Cluster-randomized design for household survey	Thematic analysis
12	Tripathy et al. [12]	2019	6 districts in three states of India	Sequential mixed-methods study	Healthcare providers and persons with diabetes at public health facilities	30 health facilities, 42 physicians, and 37 patients	Semistructured interviews, hospital records review, and observation checklist	Convenience sampling	Thematic/content analysis
13	Faruqui et al. [20]	2019	India	Qualitative study	Caregivers of children with cancer	39	In-depth interviews	Purposive sampling	Thematic content analysis
14	Jayakumar al. [25]	2019	Bihar and Jharkhand, India	Qualitative study	Female patients with visceral Leishmaniasis, health staff, local health providers, and community elders	33	Semistructured in-depth interviews	Purposive sampling	Thematic analysis
15	Kung et al. [32]	2019	Surat, India	Qualitative study	Women living with HIV	40	In-depth interviews	Purposive sampling	Content analysis
16	Holloway et al. [11]	2020	Rural India	Ethnographic qualitative study	Caregivers of sick children attending the pediatric outpatient department	43	Unstructured observations, structured observations, and semistructured interviews	Purposive sampling	Thematic analysis
17	George et al. [13]	2020	Attapadi, Kerala	Qualitative study	Indigenous community, healthcare providers, and key informants in Attapadi	47 in-depth interviews, 6 focus group discussions	Interviews, FGDs, and participant observation	Theoretical sampling	Thematic analysis
18	Raizada et al. [15]	2020	India	Mixed-method study	Pediatric tuberculosis (TB) patients	100	Semistructured questionnaires	Random selection of patients from four Indian cities	Thematic content analysis
19	Kathrikolly et al. [17]	2020	Coastal Karnataka, India	Qualitative study	Rural women aged 20-60 years	44	FGDs	Purposive sampling	Thematic analysis
20	Boro and Saikia [18]	2020	Assam, India	Qualitative study	Tribal men and women aged 25–50 residing in the study setting; health service providers from rural public health facilities	60	Semistructured open-ended questionnaire	Snowball sampling	Thematic analysis
21	Santalahti et al. [19]	2020	Manipal, Karnataka, India	Qualitative study	Migrant construction workers	15	Semistructured interviews	Convenience and purposive sampling	Thematic/content analysis
22	Wood et al. [23]	2020	India	Qualitative study	Lay mental health workers (LHWs) and stakeholders	50	In-depth interviews	Maximum variation sampling	Thematic analysis
23	Mukerji and Turan [30]	2020	Kolkata, India	Qualitative study	Female TB patients	20	In-depth interviews	Purposive sampling	Thematic analysis
24	Faruqui et al. [33]	2020	India	Qualitative study	Healthcare providers	27	Semistructured interviews	Purposive sampling	Thematic/content analysis
25	Dsouza et al. [39]	2020	India (Himachal Pradesh, Meghalaya, and Karnataka)	Qualitative study	Women aged 30-59, state/district program managers, and other healthcare staff	18	FGDs and in-depth interviews	Convenience sampling	Thematic analysis
26	Vijayan et al. [14]	2021	Family Health Center, Mundur, Thrissur district, Kerala, India	Qualitative study	Patients, medical officers, health inspectors, accountants, ASHAs, and community members	35	In-depth interviews and focus group discussions	Convenience sampling	Thematic analysis
27	Kulkarni et al. [16]	2021	Pune, Maharashtra, India	Qualitative study	Transgenders and commercial sex workers	24	In-depth interviews and focus group discussions	Purposive sampling	Thematic analysis
28	Pati et al. [42]	2021	Urban primary healthcare centers in Bhubaneswar city, Odisha, India	Qualitative study	Primary care physicians	17	Digitally recorded interviews	Purposive and convenience sampling	Thematic analysis
29	Adsul et al. [24]	2022	Mysore, India	Qualitative study	Physicians (primary care physicians, obstetricians/gynecologists, oncologists, and pathologists)	30	Semistructured interviews	Convenience sampling followed by snowball sampling	Thematic analysis
30	Wani et al. [26]	2022	North India	Mixed-method study	Healthcare workers	113	Semistructured questionnaire, interviews, and FGDs	Convenience sampling	Thematic analysis
31	Thiagesan et al. [29]	2022	Tamil Nadu, India	Qualitative study	Primary healthcare providers	13	In-depth interviews	Purposive sampling	Thematic analysis
32	Mohanraj et al [35]	2022	Uttar Pradesh (UP) and Madhya Pradesh (MP), India	Qualitative study	Healthcare providers (HCPs), including doctors and community health workers (CHWs)	23	Semistructured interviews (SSIs) and FGDs	Purposive sampling	Thematic analysis
33	Rajbangshi et al. [37]	2022	India	Qualitative study	Internally displaced Bru women in India	54	FGDs and in-depth interviews	Purposive sampling	Thematic/content analysis
34	Cáceres et al. [28]	2023	Tening, a block in Peren district, Nagaland, India	Mixed-methods study	Indigenous tribal community (Liangmai Naga tribe)	166	Semistructured cross-lingual FGDs, IDIs, and an extensive district-level community-based cross-sectional survey	Purposive sampling	Thematic analysis
35	Bangar et al. [34]	2023	Rural sites in Maharashtra, Odisha, MP, and UP	Qualitative study	Men having sex with men (MSM)	48	Interviews (key informant interviews (KIIs) and in-depth interviews (IDIs)) and FGDs	Purposive and convenient sampling	Thematic analysis
36	Saha [41]	2023	Lakhimpur district, Assam, India	Mixed-methods study	Households in flood-prone areas	83	Interview schedules	Random sampling technique	Thematic analysis

Key Findings

Facilitators of accessing and utilizing government and private healthcare facilities in India: Notable themes include care accessibility, facility cleanliness, information sharing, and staff behavior. The frequency of specific subthemes like communication clarity and physical access ease underscored their significance in patient care and healthcare delivery, indicating their critical role in service utilization (Table 2).

**Table 2:** Themes, subthemes, and codes that emerged after analysis related to facilitators of accessing government or private healthcare facilities in India One article reported only barriers.

S. No.	Author (First author)	Year of publication	Themes	Subthemes	Codes
1	Bhattacharyya et al. [38]	2015	1. Accessibility of care	1. Ease of physical access	1. Physical proximity to health facilities
			2. Cleanliness of facilities	2. Maintenance of facility hygiene	2. Regular cleaning routines
			3. Interpersonal behavior of staff	3. Respectful treatment by healthcare staff	3. Staff-patient interactions
			4. Information sharing	4. Clarity and frequency of communication with patients	4. Details of care provided and patient education
2	Varghese et al. [46]	2015	Attitude and availability of healthcare provider	Availability of local healthcare providers	1. Kindness of healthcare staff
					2. Willingness to treat common diseases
3	Gawde et al. [44]	2016	1. Social support in the home town	1. Strong social networks back home	1. Assistance from family and neighbors
			2. Familiarity with the healthcare system	2. Previous positive experiences with healthcare services	2. Recommendations from friends and neighbors
			3. Perceived quality of healthcare	3. Preference for healthcare services in Mumbai due to better infrastructure	3. Availability of services like ambulance
4	Vidler et al. [45]	2016	1. Government programs	1. Cash incentives for antenatal care visits	1. Antenatal care attendance
			2. Community health workers' encouragement	2. Institutional deliveries	2. Delivery in health facilities
			3. Availability of maternity services	3. Transport services	3. Postpartum care for neonates
5	Merugumala et al. [21]	2017	1. Community and social support	1. Peer support among parents	1. Specific instances of community help
			2. Awareness and information sharing	2. Role of healthcare professionals in education	2. Educational insights
			3. Accessibility of healthcare services	3. Charitable services providing free access	3. Financial aid instances
6	Nielsen et al. [22]	2017	1. Awareness and knowledge about GDM	1. Community outreach programs	1. Health education sessions
			2. Support systems	2. Influence of family, especially husband's support	2. Family involvement in healthcare
			3. Accessibility of Healthcare Services	3. Role of healthcare providers in educating patients	3. Doctor-patient communication
				4. Availability of screening facilities close to home	4. Ease of access to health facilities
7	Vellakkal et al. [43]	2017	1. Awareness and support	1. Awareness among women and family	1. Education on the benefits of institutional delivery
			2. ASHA (Accredited Social Health Activists) role	2. ASHA's support and services	2. ASHA's assistance in registration and transport
			3. Institutional trust	3. Trust in healthcare facilities	3. Preference for hospital due to perceived safety
8	Rath et al. [27]	2018	1. Awareness and knowledge	1. Role of mass media and education	1. Influence of educational programs
			2. Social influence and support	2. Family and community encouragement	2. Support from family members for seeking care
			3. Personal health concerns	3. Recognition of symptom severity	3. Patients' realization of the seriousness of symptoms
			4. Accessibility to healthcare services	4. Availability of local healthcare facilities	4. Ease of access to local clinics or hospitals
9	Patel et al. [31]	2018	1. Government interventions	1. Introduction of ASHAs and free health services	1. ASHA involvement
			2. Cultural shifts in health practices	2. Acceptance of institutional deliveries	2. Free service utilization
			3. Information dissemination and support services	3. Role of government schemes like Janani Shishu Suraksha Karyakram (JSSK)	3. Institutional delivery preference
10	Siddaiah et al. [36]	2018	1. Awareness and knowledge about maternal health services	1. Understanding of local health systems	Absence of knowledge gaps, awareness levels, and use of government health benefits
			2. Accessibility of public health services	2. Engagement with public health initiatives	
			3. Utilization of government schemes		
11	Elias et al. [40]	2018	1. Community engagement and awareness	1. Educational programs on non-communicable diseases (NCDs)	1. Initiatives for program awareness
			2. Accessibility of healthcare services	2. Proximity of healthcare facilities	2. Patient satisfaction with healthcare proximity
12	Tripathy et al. [12]	2019	1. Availability of resources	1. Availability of drugs and laboratory investigations	1. Availability of diagnostic tools
			2. Health system infrastructure	2. Tertiary and secondary care facilities' role	2. Provision of medications
					3. Presence of specialized consultations and diagnostic capabilities
13	Faruqui et al. [20]	2019	1. Community and family support network	1. Emotional and practical support from extended family and community	1. Moral and emotional encouragement
			2. Access to information and healthcare awareness	2. Clarity and understanding of cancer diagnosis and treatment processes	2. Acquiring and sharing knowledge about child’s condition and care options
14	Jayakumar al. [25]	2019	1. Awareness of disease	1. Health education campaigns	1. Effective community outreach
			2. Community engagement	2. Role of local health workers	2. Community advice for seeking care
			3. Government intervention	3. Insurance coverage campaign	3. Government health campaigns
15	Kung et al. [32]	2019	1. Awareness and knowledge about cervical cancer and screening	1. Understanding of cervical cancer risks and screening benefits	1. Seeking information
			2. Motivation for health maintenance	2. Health consciousness and proactive attitude	2. Prioritizing health
			3. Family and social support	3. Spousal encouragement and family involvement	3. Support from husband and other family members
16	Holloway et al. [11]	2020	Diagnostic acceptability	1. Trust in healthcare	1. Caregivers' trust in physicians' expertise
				2. Understanding and value of diagnostics	2. Perception of hospital as a high-quality, cost-effective facility
					3. Recognition of diagnostics for accurate illness identification
					4. Appreciation of diagnostics for targeted treatment
17	George et al. [13]	2020	1. Government interventions	1. Financial protection schemes	1. Free healthcare services and reimbursement of indirect costs
			2. Community engagement	2. Healthcare infrastructure improvement	2. Upgraded health facilities and specialist appointments
				3. Inclusion in decision-making	3. Village chief consultations and community health committees
18	Raizada et al. [15]	2020	1. Proactive family support	1. Willingness to seek care	1. Family's determination for treatment
			2. Availability of diagnostic tools	2. Knowledge of TB management	2. Awareness of TB symptoms
			3. Public health interventions and policies	3. Accessibility to free diagnostic testing	3. Use of public sector facilities for testing
19	Kathrikolly et al. [17]	2020	1. Health concerns of women	1. Self-care activities	1. Awareness of breast cancer and screening
			2. Factors motivating the uptake of screening facilities	2. Awareness camps	2. Influence of female healthcare providers
			3. Perception of cancer prevention and control initiatives	3. Government-led initiatives	3. Community-based information dissemination strategies
			4. Information dissemination	4. Mass media and interactive methods for information sharing	4. Involvement of men in promoting women’s health
			5. Role of men in women’s health	5. Educating men on women's health	
20	Santalahti et al. [19]	2020	1. Awareness and education	1. Role of healthcare workers	1. Knowledge about breast cancer
			2. Supportive healthcare providers	2. Community-based health programs	2. Trust in female doctors and nurses
			3. Community engagement		3. Effectiveness of health camps
21	Wood et al. [23]	2020	1. Community engagement	1. Community trust	1. Instances of positive community interactions
			2. Training and support	2. Comprehensive training	2. Supportive family anecdotes
			3. Personal growth	3. Peer support	3. Effective training experiences
				4. Self-efficacy	4. Positive patient outcomes
22	Mukerji et al. [30]	2020	1. Supportive clinic environment	1. Positive staff-patient interaction	1. Staff encouragement
			2. Free medication provision	2. Financial relief through no-cost TB treatment	2. Absence of treatment fees
23	Faruqui et al. [33]	2020	1. Integrated healthcare infrastructure	1. Enhanced facility resources	1. Availability of specialized equipment
			2. Comprehensive training programs	2. Professional development and education	2. Skill enhancement initiatives
			3. Multidisciplinary care approach	3. Collaboration across specialties	3. Team-based patient management
24	Dsouza et al. [39]	2020	1. Awareness and education	1. Community engagement and outreach programs	1. Health education sessions
			2. Accessibility of services	2. Mobile health units and local health camps	2. Availability of nearby health facilities
			3. Social support and encouragement	3. Role of family and community leaders in promoting healthcare utilization	3. Encouragement from family members
25	Vijayan et al. [14]	2021	1. Good staff behavior	1. Enhanced patient-healthcare provider relationship	1. Patient-staff interaction
			2. Evening outpatient services	2. Increased healthcare access for working people and school children	2. Operational hours
			3. Specialty clinics	3. Benefits to the geriatric population	3. Geriatric services
			4. Improved infrastructure	4. Patient satisfaction with the health center's environment	4. Infrastructure quality
			5. Accessibility	5. Convenience of accessing the health center	5. Accessibility and parking
			6. Subsidized laboratory services	6. Motivation for staff	6. Laboratory affordability
			7. Local self-government involvement		7. Governmental support
			8. Rewards		8. Performance incentives
26	Kulkarni et al. [16]	2021	1. Awareness and knowledge	1. Understanding of eye care needs	1. Recognition of visual impairment
			2. Community support	2. NGO and social worker involvement	2. Knowledge of available treatments
			3. Healthcare provider efforts	3. Sensitivity and inclusivity	3. Assistance in accessing healthcare
					4. Advocacy and guidance
					5. Efforts to provide gender-neutral facilities
					6. Empathetic treatment by some healthcare providers
27	Pati et al. [42]	2021	1. Health system support	1. Availability of NCD awareness campaigns	1. Periodic follow-ups by health workers
			2. Professional development and training	2. Support from pharmaceutical companies	2. Provision of updated treatment modalities
			3. Community engagement and awareness	3. Formal training in diabetes management	3. Confidence in handling diabetes with comorbidities
				4. Peer learning and networking	4. Use of seminars and workshops for knowledge enhancement
				5. Role of community health workers	5. Active engagement in treatment plans
				6. Patient education campaigns	6. Increased patient attentiveness to treatment
28	Adsul et al. [24]	2022	1. Community engagement	1. Culturally-tailored education	1. Peer-led educational programs
			2. Provider training	2. Utilization of community health workers	2. Integration with women's social groups
			3. Health literacy enhancement	3. Physician training programs	3. Physician readiness for screening procedures
29	Wani et al. [26]	2022	1. Supportive institutional policies	1. Availability of a dedicated palliative care unit	1. Institutional support
			2. Training and education	2. Regular training programs	2. Professional development
					3. Policy amendments
30	Thiagesan et al. [29]	2022	1. Innovative care approaches	1. Local health innovations	1. Use of physical activity adaptations
			2. Community and peer support	2. Formation of peer support groups	2. Peer education and support activities
				3. Cross-program resource utilization	3. Integration of health services
31	Mohanraj et al. [35]	2022	1. Strengthening public health infrastructure	1. Improved availability and quality of public health facilities	1. Infrastructure improvements
			2. Enhancing community health literacy	2. Enhanced trust in government healthcare services	2. Trust in public health services
			3. Effective utilization of community health workers	3. Increased awareness about childhood pneumonia symptoms and treatment	3. Awareness programs
				4. Education on the importance of seeking timely and appropriate care	4. Training programs for community health workers
				5. Regular training and skill enhancement of community health workers	5. Community health workers support mechanisms
				6. Support and supervision for community health workers	
32	Rajbangshi et al. [37]	2022	1. Community health worker engagement	1. Role of ASHAs in healthcare delivery	1. ASHAs assisting with institutional deliveries
			2. Awareness of health services	2. Knowledge of reproductive health options	2. Awareness of temporary contraceptive methods
				3. Positive attitudes toward seeking care	3. Knowledge about medical abortion
					4. Willingness to use reproductive and maternal health (RMH) services
33	Cáceres et al. [28]	2023	1. Availability of healthcare services	1. Proximity of healthcare facilities	1. Nearby health centers
			2. Community support	2. Role of family and neighbors	2. Family encouragement
			3. Awareness programs	3. Government health campaigns	3. Health education sessions
34	Bangar et al. [34]	2023	1. Community engagement and sensitization	1. Role of community liaison officers	1. Empathetic healthcare providers
			2. Provision of specialized healthcare services	2. Need for MSM-specific health workers	2. Peer counseling
			3. Confidentiality and privacy in healthcare	3. Safe spaces for health discussions	3. Discreet health service locations
35	Saha et al. [41]	2023	1. Availability of boat clinics	1. Mobile health clinics as essential services	1. Use of boat clinics for healthcare
			2. Community resilience and adaptation	2. Community support systems	2. Community-led health initiatives

Predominant Themes and Codes

The analysis revealed 10 central themes impacting healthcare access and utilization (Table 3).

**Table 3:** Identified top themes and codes for the facilitators of accessing healthcare facilities

Theme	Description
Information sharing	Communication and health education
Cleanliness of facilities	Maintaining clean healthcare environments
Staff-patient interactions and empathy	Positive interactions, empathy, and compassionate care
Physical accessibility and proximity	Geographical accessibility to healthcare facilities and proximity to patients’ residences
Patient education and awareness	Educating patients about conditions, treatments, and available healthcare services
Knowledge of treatment options	Awareness of different treatment modalities and options
Gender-sensitive facilities	Facilities designed for diverse genders and inclusivity
Assistance programs and community support	Community-based assistance, support networks, and outreach programs
Advocacy and guidance	Efforts to bridge gaps in healthcare access through advocacy
Visual impairment considerations	Addressing the needs of visually impaired individuals within healthcare settings
Patient-centered care and trust-building	Shared decision-making, trust-building, and patient empowerment

Verbatim quotes (along with brief explanations) representing various facilitators are presented under the most frequently occurring codes and prominent themes (Table 4).

**Table 4:** Verbatim quotes representing various facilitators FHC: Family health centers.

Facilitator themes	Verbatim quotes
Accessibility of care [14,22,27,28,38,39,40,44]	Accessibility of care encompasses the availability of healthcare services considering geographical, financial, and logistical factors. Studies stress the importance of proximity to healthcare facilities, particularly for low-income groups favoring government facilities. Local facility access is recognized as a facilitator. Qualitative research in central Kerala highlighted ease of access and ample parking as key factors influencing healthcare utilization [14]. Financial affordability considers direct costs, insurance coverage, and subsidies. Logistical accessibility involves transportation, appointment scheduling, and wait times. One of the patients in a FGD in a studyquoted [14]: "The hospital has changed. We don't have to stand in a queue. There are chairs to sit while we wait for our turn in the doctor's consultation room and in the pharmacy” (patient, FGD).
Interpersonal behavior of staff [14,16,20,22,28,34,38,42,46]	Empathetic treatment and positive staff-patient interactions emerged as prominent facilitators in healthcare access and utilization. Studies highlighted instances where healthcare providers showed understanding and empathy, fostering trust and comfort. Promoting such positive interactions is crucial for motivating community members to utilize family health center services, as found in a qualitative study conducted in central Kerala [14]. A patient in an FGD in this study said, “All the staff here has good behaviour to patients and they are ready to listen to what we want to say.” In another study [28], a woman said, “The health staff and nurses all take good care (.) They are all from our village. Even the doctor is from our own Liangmai tribe. I can communicate well and share all my problems in my own language, which is very good”—Liangmai woman, 26 years old. In a study [38],one user (of healthcare) quoted “Nurse and dai were very supportive. They told me not to worry and stay calm.” The significance of staff-patient interaction through emotional support was pointed out in a study[38]. A nursing staff in the same study commented, “As soon as the woman enters, we make her to lie down comfortably. We console her that we will be there with her for all support… whatever difficulties arise, we will handle them. We try to keep the woman continue talking even while in pain and we will do our work simultaneously.”
Cleanliness of facilities [38]	Reflects the critical nature of cleanliness and sanitation in healthcare settings for infection control and patient safety. It points to the fundamental role of hygiene in patient care and the prevention of hospital-acquired infections. In a study, cleanliness was pointed out as a major challenge both by users and providers of healthcare, and if addressed, could easily facilitate healthcare access and utilization [38]. "They (sweeper) clean the ward and toilet once a day. But the toilet is dirty most of the time. Sometimes, women do not pour enough water. Water supply is also not regular"—comment by a ward nurse [38]. "Our staff take regular care in cleaning wards and toilets. However, these village people do not know how to maintain cleanliness. They make the place dirty"—comment by a doctor [38].
Community support [13,14,16,28,29]	Advocacy and guidance: The role of community support networks in facilitating healthcare access and utilization emerged in some studies. Community leaders or organizations might help navigate healthcare systems, particularly in rural areas. In a study [14], the fact that local self-government was actively involved in the functioning of the health center was considered a facilitator. An accountant commented, “We are receiving added funds since we became an FHC, now we are able to utilize these funds for a lot of improvements in the center.” It was one of the most frequently identified facilitators, indicating that active support and guidance for patients are crucial. It highlights the importance of navigational assistance in the healthcare system and the positive impact of advocacy programs on healthcare access.
Efforts to provide gender-neutral facilities and recognition of visual impairment [16]	Points to the specific acknowledgment and accommodation of visual disabilities in healthcare settings. It emphasizes the need for tailored healthcare services that address the unique challenges faced by visually impaired patients. Some studies highlighted efforts by private facilities to create gender-neutral restrooms or waiting areas, facilitating access for individuals with diverse gender identities. For instance, the comment of a 60-year-old transgender person was considered a barrier to healthcare utilization: “There are only male or female wards in the hospital. Where do people like me stay? Other patients would abuse me. Therefore, I am avoiding cataract surgery.” In this study [16], gender-specific challenges were recommended to be addressed to facilitate access to quality healthcare services. In the same study[16], it was stated that all individuals with severe visual impairment (SVI) or blindness, including transgender individuals (TGs) and commercial sex workers (CSWs), were provided with free treatment and rehabilitation services, irrespective of their involvement in the study.
Information sharing [19,20,21,22,37,38,39]	In the analysis, it was consistently observed that details of care provided and patient education played crucial roles. Clear communication regarding treatment options, procedures, and discharge instructions emerged as significant facilitators in ensuring access to healthcare services. Notably, users emphasized the importance of receiving timely and comprehensive information from healthcare providers, particularly regarding tests and their schedules [22]. Addressing this need for information sharing could greatly enhance access to quality healthcare services [38].
Healthcare provider efforts [16,19]	Assistance in accessing healthcare emerged as a dominant facilitator code, highlighting the importance of logistical support in overcoming barriers. This underscores the value of transportation, financial aid, and appointment scheduling help.
Knowledge of available treatments [16,19,37]	Awareness of treatment options is pivotal for patients, underscoring the necessity of clear communication between healthcare providers and patients. Instances in government healthcare settings where providers exhibit sufficient knowledge of available treatments facilitate access to appropriate care within the public healthcare system. Health system support [16,19,37], professional development and training, and community engagement emerged as top facilitators. In support of the above factors, in a study [37], an awareness campaign that included information, education, and communication (IEC) on noncommunicable diseases (NCDs) in the community and health facilities was perceived as a facilitator. The practitioners in a mixed-method study [23] emphasized a holistic palliative care approach, which included training for doctors and healthcare workers as part of a comprehensive package. Further investigation could reveal additional facilitators specific to demographics like people with disabilities, focusing on accessibility enhancements such as ramps or assistive devices.

The key codes are depicted in the word cloud. The main words getting repeated are "awareness," "treatment," "encouragement," etc. (Appendices).

Barriers to Accessing and Utilizing Government and Private Healthcare Facilities in India

Barriers uncovered that influenced individuals' ability to access and utilize healthcare services included a shortage of manpower and equipment, poor transport, and cultural norms (Table 5).

**Table 5:** Themes, subthemes, and codes that emerged after analysis related to barriers of accessing government or private healthcare facilities in India

S. No.	Author (First author)	Year of publication	Themes	Subthemes	Codes
1	Bhattacharyya et al. [38]	2015	1. Inadequate infrastructure	1. Insufficient facilities and equipment	1. Specific infrastructural deficits
			2. Supply shortages	2. Irregular availability of water, electricity, and medicines	2. Items frequently out of stock
			3. Staffing issues	3. Shortage of gynecologists and anesthetists	3. Specific staffing gaps
			4. Privacy concerns	4. Difficulty in maintaining patient confidentiality	4. Examples of privacy violations
			5. Lack of post-delivery counseling	5. Inadequate training in post-delivery care	5. Lack of counseling sessions
			6. Management of referral cases	6. Challenges in handling emergency referrals	6. Case studies of referral management
			7. Nonfunctional blood banks	7. Operational issues in blood banks	7. Instances of blood bank failures
			8. Lack of incentives for staff	8. Demotivation among healthcare workers due to inadequate rewards	8. Expressions of staff dissatisfaction
2	Varghese et al. [46]	2015	1. Financial	1. High treatment costs	1. Cost of treatment
			2. Structural	2. Unawareness of government aid	2. Inaccessibility of specialists
			3. Cognitive	3. Poor availability of healthcare providers	3. Long distances
				4. Poor transportation difficulties	4. Misunderstanding of the condition
				5. Lack of referrals	5. Lack of preventive care
				6. Lack of caregiver knowledge	6. Inadequate training
				7. Inadequate provider education	
3	Gawde et al. [44]	2016	1. Sociocultural factors	1. Lack of felt need for healthcare	1. Preference for home delivery due to cost and cultural practices
			2. Economic constraints	2. High living and healthcare costs in Mumbai	2. Difficulties in navigating the urban healthcare system
			3. Structural barriers	3. Inadequate health infrastructure	3. Limited access to government programs for migrants
			4. Health system challenges	4. Cumbersome administrative procedures	4. Lack of migrant-friendly healthcare policies
4	Vidler et al. [45]	2016	1. Limited autonomy	1. Cultural beliefs	1. Decision-making power of women
			2. Poor access to transport	2. Availability and cost of transport	2. Access and affordability of transport
			3. Perceived poor quality of healthcare facilities	3. Maintenance of health facilities	3. Satisfaction with health services
			4. Financial constraints	4. Costs related to healthcare	4. Direct and indirect costs of care
5	Merugumala et al. [21]	2017	1. Cultural beliefs	1. Influence of elders	1. Specific beliefs of family elders
			2. Socioeconomic challenges	2. Financial constraints	2. Instances of financial hardship
			3. Healthcare system limitations	3. Transportation difficulties	3. Travel difficulties to healthcare centers
6	Nielsen et al. [22]	2017	1. Healthcare system challenges	1. Inadequate healthcare facilities	1. Lack of resources at health centers
			2. Sociocultural factors	2. Stigma and societal norms affecting health-seeking behavior	2. Social stigma related to GDM
			3. Personal barriers	3. Personal constraints like time and financial issues	3. Work and family commitments
					4. Economic constraints
					5. Out-of-pocket expenses
					6. Long wait time at clinics
7	Vellakkal et al. [43]	2017	1. Sociocultural norms	1. Cultural preference for home delivery	1. Belief in natural childbirth
			2. System and environmental limitations	2. Distance to facilities and poor quality of care	2. Negative past experiences with healthcare facilities
			3. Financial and opportunity costs	3. High costs of hospital delivery versus the cash incentive	3. Perceived lower benefit of the cash incentive compared to the costs and efforts involved
8	Rath et al. [27]	2018	1. Ignorance and negligence	1. Lack of symptom recognition	1. Disregarding early symptoms as minor issues
			2. Financial constraints	2. Economic difficulties in affording treatment	2. Inability to pay for medical expenses
			3. Health system challenges	3. Inefficiency and delays in the healthcare system	3. Experiences of misdiagnosis or delayed diagnosis
			4. Cultural beliefs and stigma	4. Fear of stigma	4. Concerns about being stigmatized due to illness
9	Patel et al. [31]	2018	1. Inadequate health services infrastructure	1. Insufficient ASHA (Accredited Social Health Activists) coverage and unauthorized charges	1. Limited health workforce
			2. Financial constraints	2. Costs related to transport and unofficial payments	2. Financial burdens and unofficial costs
			3. Information and awareness gaps	3. Lack of knowledge about available services and emergency care	3. Emergency care awareness and information deficiency
10	Siddaiah et al. [36]	2018	1. Misconceptions and mistrust about the public health system	1. Influence of substandard private healthcare at brick kilns	Mistrust, misinformation, inaccessibility, systemic apathy, and partial insurance coverage
			2. Barriers to Universal Health Coverage	2. Systemic issues in public healthcare	
			3. Socioeconomic and logistical challenges	3. Geographical and time-related constraints	
11	Elias et al. [40]	2018	1. Systemic healthcare deficiencies	1. Inadequate healthcare infrastructure	1. Reports of healthcare worker shortages
			2. Financial constraints	2. High out-of-pocket expenditures	2. Burden of medical costs on families
			3. Social and cultural barriers	3. Stigma and discrimination in healthcare settings	3. Negative perceptions of programs
12	Tripathy et al. [12]	2019	1. Systemic and structural challenges	1. Insufficient resources at primary level	1. Lack of diagnostic tools at primary health centers
			2. Operational and management issues	2. Patient overload and lack of training	2. Overcrowding at tertiary facilities
			3. Patient-centered care challenges	3. Accessibility and follow-up	3. Lack of specialized diabetic care training for primary healthcare providers
					4. Difficulty in patient follow-up due to system limitations
					5. Lack of patient education and counseling services
13	Faruqui et al. [20]	2019	1. Healthcare system limitations	1. Complex referral systems and inadequate medical facilities	1. Navigating multiple medical appointments and tests
			2. Economic hardships	2. Financial burdens due to treatment and associated costs	2. Struggles with funding treatment and associated travel
			3. Emotional distress and mental health concerns	3. Psychological impact on families and caregivers	3. Stress, anxiety, and helplessness experienced by caregivers
14	Jayakumar al., [25]	2019	1. Sociocultural norms	1. Gender roles in healthcare decisions	1. Need for male accompaniment
			2. Economic constraints	2. Financial barriers to seeking treatment	2. Cost of private healthcare
			3. Healthcare accessibility	3. Distance to healthcare facilities	3. Travel difficulties
			4. Perception of care quality	4. Distrust in government hospitals	4. Long wait times at government facilities
15	Kung et al. [32]	2019	1. Lack of knowledge and awareness	1. Misunderstanding about cervical cancer and its screening	1. Information gap
			2. Fear and apprehension toward screening	2. Fear of screening procedure and results	2. Anxiety about medical procedures
			3. Limited family and social support	3. Lack of encouragement or assistance from family	3. Absence or indifference of family support
16	Holloway et al. [11]	2020	Barriers to diagnostic utilization	1. Navigational challenges	1. Inadequate infrastructure for diagnostics
				2. Delays and necessity for return visits	2. Lack of point-of-care testing
				3. Cost of diagnostic tests	3. Economic feasibility concerns for patients and hospitals
				4. Additional expenses (travel and lost wages)	4. Economic barriers for the poorest families
					5. Balancing healthcare needs with work and household responsibilities
					6. Navigational challenges for uneducated or illiterate caregivers
					7. Perceptions about the necessity of diagnostics
					8. Prioritizing symptomatic treatment over diagnostic tests
17	George et al. [13]	2020	1. Knowledge and awareness gaps	1. Marginalization of indigenous practices	1. Disregard for traditional medicine and lack of culturally respectful care
			2. Centralization of healthcare	2. Fear and mistrust	2. Fear of hospitalization and mistrust in modern healthcare
			3. Discrimination and Stigma	3. Accessibility challenges	3. Travel difficulties and overcrowded central hospitals
			4. Limited community empowerment	4. Bias in healthcare provision	4. Discriminatory attitudes and unconscious bias
			5. Socioeconomic challenges	5. Exclusion from policy making	5. Lack of genuine involvement and tokenistic inclusion
				6. Land rights and livelihood	6. Loss of traditional lands and impact on nutrition and health
18	Raizada et al. [15]	2020	1. Diagnostic delays	1. Complexity of TB diagnosis in children	1. Multiple healthcare provider visits before diagnosis
			2. Socioeconomic challenges	2. Costs associated with healthcare seeking	2. Financial burden of TB treatment
			3. Stigma and social isolation	3. Fear of social ostracization	3. Families hiding the TB diagnosis to avoid stigma
19	Kathrikolly et al. [17]	2020	1. Sociocultural constraints	1. Cultural inhibitions	1. Fear and stigma related to cancer
			2. Economic challenges	2. Financial burden	2. Financial constraints for screening
			3. Health system limitations	3. Infrastructure gaps	3. Accessibility issues of mammogram centers
					4. Presence of male health workers as a deterrent
20	Boro et al. [18]	2020	1. Affordability	1. Direct and indirect costs	1. Economic hardship
			2. Quality of care	2. Availability of healthcare providers	2. High treatment costs
			3. Infrastructure and accessibility	3. Interpersonal care quality	3. Transportation costs
			4. Cultural and educational influences	4. Medicine and equipment availability	4. Doctor availability
				5. Traditional medicine preference	5. Long waiting times
				6. Educational limitations	6. Disrespectful treatment
				7. Geographical barriers	7. Lack of essential drugs
				8. Government and institutional support	8. Diagnostic services
					9. Educational gaps
					10. Distance challenges
					11. Overburdened facilities
					12. Noncompliance and misconceptions
21	Santalahti et al. [19]	2020	1. Financial constraints	1. Unsteady employment	1. Daily wage dependency
			2. Structural and accessibility issues	2. High medical costs	2. Lack of savings, debt for medical expenses
			3. Social and cultural barriers	3. Lack of awareness about health services	3. Unfamiliarity with healthcare system
			4. Policy and documentation challenges	4. Distance to health facilities	4. Transportation challenges
			5. Work-related limitations	5. Discrimination in healthcare settings	5. Prejudice against migrants
				6. Language barriers	6. Communication difficulties due to language
				7. Low education levels leading to unawareness or misconceptions	7. Inability to present identification
				8. Lack of proper documentation	8. Exclusion from government health schemes
				9. Inflexible work hours	9. No time off for medical visits
22	Wood et al. [23]	2020	1. Stigma	1. Societal stigma toward mental health	1. Instances of community resistance
			2. Resource limitations	2. Lack of resources and support	2. Challenges in patient engagement
			3. Role ambiguity	3. Lack of professional recognition	3. Feelings of isolation and burnout among healthcare workers
23	Mukerji et al. [30]	2020	1. Systemic healthcare challenges	1. Inadequate healthcare infrastructure	1. Overcrowded clinics
			2. Sociocultural factors	2. Stigma and privacy concerns	2. Fear of social exclusion
			3. Economic constraints	3. Direct and indirect costs of treatment	3. Transportation and nutritional costs
24	Faruqui et al. [33]	2020	1. Systemic healthcare limitations	1. Inadequate public health facilities	1. Resource scarcity in public hospitals
			2. Socioeconomic constraints	2. Financial burden on families	2. Out-of-pocket expenses for treatment
			3. Cultural and societal influences	3. Stigma and lack of awareness	3. Misconceptions about cancer treatment
25	Dsouza et al. [39]	2020	1. Sociocultural stigmas and beliefs	1. Cultural resistance to modern healthcare	1. Misconceptions about diseases and treatments
			2. Systemic and organizational challenges	2. Inadequate health infrastructure and staffing	2. Long waiting times and lack of privacy
			3. Individual perceptions and fear	3. Fear of diagnosis and treatment	3. Anxiety regarding medical procedures
26	Vijayan et al. [14]	2021	1. Staff shortage and workload	1. Operational challenges due to staff shortage	1. Staffing issues
			2. Lack of awareness among the population	2. Public unawareness of certain health services	2. Public knowledge gaps
			3. Shortage of medicines	3. Disruptions in medicine supply affecting chronic disease management	3. Medicine availability
27	Kulkarni et al. [16]	2021	1. Social stigma and discrimination	1. Societal attitudes	1. Stigmatization in public facilities
			2. Financial challenges	2. Economic hardships	2. Fear of discrimination from healthcare providers
			3. Mental health concerns	3. Psychological barriers	3. Need to hide identity
			4. Systemic and structural issues	4. Healthcare system limitations	4. Poverty
			5. Personal beliefs and attitudes	5. Individual perceptions	5. Lack of stable income
					6. Financial prioritization away from health
					7. Depression
					8. Emotional distress
					9. Impact on health-seeking behavior
					10. Lack of gender-neutral facilities
					11. Language barriers
					12. Legal and documentation challenges
					13. Myths and superstitions
					14. Fear of seeking healthcare
28	Pati et al. [42]	2021	1. Systemic challenges	1. Infrastructure and logistics	1. Irregular medicine supply
			2. Physician-related barriers	2. Management of records and documentation	2. Poor laboratory services
			3. Patient-related factors	3. Lack of formal training and skills	3. Lack of skilled support staff
				4. High patient density and inadequate time	4. Overreliance on self-education through the internet and pharmaceutical companies
				5. Socioeconomic status	5. Communication difficulties with patients
				6. Treatment adherence	6. Mistrust in government-supplied medicines
					7. Noncompliance with treatment plans due to traditional beliefs
29	Adsul et al. [24]	2022	1. Cultural stigma	1. Sociocultural barriers to discussing gynecologic health	1. Women’s health as a low priority
			2. Resource limitations	2. Women’s autonomy in healthcare decisions	2. Inadequate training for cervical cancer screening among physicians
			3. Lack of awareness	3. Costs related to healthcare access	3. Misconceptions about screening
				4. Community myths and fears about cancer	
30	Wani et al. [26]	2022	1. Resource constraints	1. Inadequate staffing	1. Untrained staff
			2. Lack of awareness	2. Limited awareness among healthcare workers	2. Knowledge gap
					3. Medicine shortage
31	Thiagesan et al. [29]	2022	1. Accessibility challenges	1. Poor physical access to healthcare facilities	1. Dependency on others for daily activities
			2. Treatment compliance difficulties	2. Difficulties in adopting recommended dietary and physical activity changes	2. Inaccessibility of higher-level healthcare facilities for annual screenings
			3. Lack of tailored health guidelines	3. Lack of standardized guidelines for diabetes management in persons with disabilities	3. Healthcare providers' reliance on medication prescription due to the inability to offer comprehensive care
32	Mohanraj et al. [35]	2022	1. Inadequate health infrastructure	1. Lack of essential drugs and medical equipment	1. Equipment and drug shortages
			2. Community practices and perceptions	2. Insufficient healthcare personnel	2. Staff shortages
			3. Skill deficits among health workers	3. Delayed care-seeking behavior	3. Poor community awareness
				4. Preference for local unqualified providers due to accessibility and trust issues	4. Reliance on unqualified providers
				5. Insufficient knowledge and skills among CHWs and paramedical staff	5. Lack of training for community health workers
				6. Lack of regular updates and refresher training for health workers	6. Supervision gaps for health workers
33	Rajbangshi et al. [37]	2022	1. Limited healthcare accessibility	1. Transportation difficulties to health facilities	1. High cost of private transportation for medical care
			2. Financial constraints	2. Cost of medical care and lack of funds	2. Inability to afford health services costs
			3. Knowledge and awareness gaps	3. Lack of awareness about comprehensive health services	3. Lack of knowledge about government health schemes and broader contraceptive options
			4. Discrimination and cultural barriers	4. Language and cultural disconnect with healthcare providers	4. Language barriers impeding communication with healthcare workers
				5. Challenges in healthcare provider attitudes	5. Perceived discrimination by healthcare providers
					6. Reluctance of health providers to serve displaced populations
					7. Poor reach of public health insurance schemes in displaced communities
					8. Challenges in opening bank accounts for receiving health scheme benefits
34	Cáceres et al. [28]	2023	1. Economic constraints	1. Affordability of services	1. Cost of care
			2. Sociocultural factors	2. Gender norms	2. Women's autonomy
			3. Systemic issues	3. Staff attitudes	3. Discrimination by healthcare workers
35	Bangar et al. [34]	2023	1. Societal stigma and discrimination	1. Fear of exposure and societal backlash	1. Non-recognition of MSM identity in healthcare
			2. Invisibility of MSM in healthcare	2. Inadequate healthcare services for MSM	2. Inadequate information and education on HIV
			3. Lack of awareness and misconceptions about HIV	3. Denial of self-risk for HIV among MSM	3. Cultural and homophobic barriers
			4. Programmatic gaps in healthcare services	4. Poor quality of government health facilities	4. Dissatisfaction with government healthcare services
36	Saha et al. [41]	2023	1. Lack of transportation facilities	1. Damaged roads and absence of reliable transport	1. Road damage and isolation
			2. Nonfunctional subcenters	2. Subcenters lacking basic facilities and healthcare personnel	2. Inadequate healthcare facilities
			3. Financial constraints	3. Economic hardships affecting healthcare access	3. Economic inability to afford care
			4. Poor infrastructure and inaccessibility during floods	4. Increased health risks due to flood conditions	4. Waterborne diseases and lack of sanitation

Predominant Themes and Codes

The study pinpointed key barriers to healthcare utilization in India, with systemic challenges being the most prevalent, followed by physician-related and patient-related barriers. Verbatim quotes along with brief explanations representing various barriers are presented under the most frequently occurring and prominent codes identified during the detailed analysis (Table 6).

**Table 6:** Verbatim quotes representing various barriers The content is taken from published literature (under a Creative Commons License). UPHC: Urban primary healthcare centers.

Barrier themes	Verbatim quotes
Specific staffing gaps [12,24,26,35,38,42,46]	This identified barrier code points to the lack of specialized healthcare personnel in certain areas, affecting service quality and patient outcomes. It indicates a need for targeted training and recruitment strategies. In a study [35], this factor was clearly stated as a barrier to access quality healthcare services. One of the studies[42] pointed out that the physicians who did not receive any training were not aware of standard clinical guidelines for the treatment of chronic conditions. In this study [35], a district health officer said, “Till now, training has been given to Anganwadi workers, ASHAs. But even after training they do not have the skills to identify pneumonia properly. At least they should be able to give treatment there and send serious cases to the hospital. We have given them the tablets-cotrimoxazole, but if they are not able to identify, how would they give the medicine correctly to the children? They do not have this knowledge though training has been given.” Quote from another study [38] also depicts the same, “16 posts are sanctioned for medical officers, but only five are filled… no pathologist and paediatrician… Every staff member is overworked. We do not get any leave. Doctors are not willing to join the government service because of low salary and poor working conditions.” Quoted by a physician [42], “they give training only on the current epidemic… yes of course, if there would have been some training on NCD… then there we could have a standardised procedure for treatment, we could have followed a protocol of state govt. By which we could sort out the problems we are facing daily.”
Specific infrastructural deficits [11,14,16,18,22,26,29,33,38,41,42]	Identifies physical inadequacies within healthcare facilities that can impede service delivery. It emphasizes the need for investment in healthcare infrastructure to provide a conducive environment for patient care. In a study [26], it was clearly stated that the lack of infrastructure/staff for the provision of palliative care was one of the major barriers to providing palliative care. In a qualitative study[38] on maternity care in secondary-level public health facilities in Uttar Pradesh, a doctor quoted, “There are 10 labour tables in the facility while the average delivery load is 20 per day. Availability of stretchers is limited and many women are shifted to labour room on foot. Trolley is not working properly…” showing a lack of infrastructure as a barrier to access healthcare in the country. “We see many patients who actually need palliative care.” “Sir, many times we get patients with end stage malignancies, end stage liver disease, patients who cannot afford 30–40 lakhs or E-S renal disease. We know that they cannot afford a renal transplant or dialysis. Hence, we want these patients to be as comfortable as possible before they die, but the problem is that we do not have a separate department or unit to provide care. At the most we tell them that this patient is not going to survive and you can take this patient home but that is not how palliative care works in my opinion” [26]. “Pain was increasing… the community health worker called the ambulance service around 11 am. The vehicle came after an hour. I reached the nearby health facility after 30 minutes. I was anaemic and had a breech baby. So they (first facility) referred me to district hospital, which was 50 km away from the first facility. The ambulance took another 1 hour to reach the district hospital.” This quote clearly shows the infrastructural deficit and its being a barrier to access healthcare services [38].
Lack of counseling sessions [16,34,38]	Reveals a gap in providing mental health support and patient counseling, which is integral to comprehensive healthcare. It suggests a need for integrating mental health services into routine care. Participants from rural areas in a study[34] voiced the need for counseling. Lack of information sharing and counseling were also highlighted as a barrier in another study [38] conducted in Uttar Pradesh. A quotein [34] states, "there the main role is of counseling. The community member whether he is kothi (sexually receptive MSM) or panthi (MSM who practice penetrative sex or both penetrative and receptive sex) or DD [/Double Decker/] (sexually receptive and penetrative MSM) whatever he is, so he [/MSM/] can share everything with the counselor freely. The counseling is the only session where he [/MSM/] can share all his views. For that we have to give them [/MSM/] a special time, we have to give the time according to their needs, we have to meet them according to their comfort." One of the women who had a normal delivery said, “They (facility staff) were giving us just instructions… buy this, bring that, do this, do that.... They never explained the reason” [38]. Another woman quoted, “Consent (for C-section) was taken on a form but they (facility staff) did not share the reason for taking the consent… nobody asked whether we wanted to do the operation or not” [38].
Medicine availability and items frequently out of stock [14,18,26,35,38,42]	The highest frequency of occurrence indicates a critical issue with essential medicine supply, highlighting challenges in procurement, distribution, and inventory management in healthcare facilities. Logistical hurdles in maintaining medical supplies hinder healthcare provision, necessitating improved supply chain management. Physicians in Odisha emphasized medicine unavailability as a significant barrier [42]. A 44-year-old man in a study [18] quoted, “Only medicines for fever, cough, and cold are available in the nearby dispensary. Most of the time, the stock of medicines runs out, and it takes 3–4 days for new medications to arrive.” In a study[35] conducted in various districts of two states of India, the community health workers in an FGD reported “We don’t have any medicines with us… If we have medicines with us, then everyone will come to us…” Another quoted “Except paracetamol, nothing is given, that too is not given sufficiently” [35]. One participant [35] raised concern by saying “In the last 6 months we never received amoxicillin tablets. Last year we received syrup once. Small bottles of 10 ml are what we get, how is it adequate?” “To the only diabetic patient we give the medicines to decrease the blood sugar level but in comorbidity we also have to treat the other diseases and sometimes we don’t have the supply of diabetes medicines... in other chronic diseases we have for acid peptic disease… blood pressure also the medicines come but periodically” [42].
Public knowledge gaps [12,14,18,26,31,32,34,35,37,42]	Indicates a lack of health literacy and awareness among the public, which can lead to delays in seeking care or mismanagement of health conditions. It stresses the need for public health education initiatives. An example of the lack of awareness (about HIV) in a study [34]supports the fact that it is a barrier to seek care. In a study [18], an auxiliary nurse midwife (ANM) working at a subcenter said, “When iron tablets are prescribed to pregnant women, they do not take them. They think it will increase the weight of the baby. Some say they get a particular smell, so they cannot take those tablets.” One of the participants in the study[34] quoted, “About HIV, I have only heard that it happens. Now I do not know how it [/HIV/] happens [/gets transmitted/] and why it happens. Because I haven’t done anything like that [/risky/] for which I have to go to see a doctor. If it was [/risky/], then I would have told you. No madam I haven’t heard. But, yes, I have read [/about/] it in the newspaper, otherwise, I haven’t heard of it. Here the venereal disease doctor comes from = Bhopal = [/Capital city, MP/], I have seen his advertisement in a newspaper. Otherwise, I had not heard of it.” This barrier was clearly stated in another study[35] in which a district health officer quoted, “There is a lack of awareness about pneumonia, specifically in remote areas plus they follow indigenous treatments like using ‘sambhar ke seeengh’ [medicine prepared from horns of deer]. Some give haldi [turmeric], some rub garlic etc. They do all such things. When the child is serious they give brandy to drink… all this is done. When condition is critical then they take to the hospital.” “People come in late and I am not in a condition to do anything at that time. Like when the child comes in gasping stage or sudden respiratory distress with only [count of], 2–4 heartbeats present. They first consult the quack [UCP]when there is no relief from quack then they try other people nearby. One more thing I have seen that people in the community give suggestion—lets go and consult so and so then child will be all right…. people roam here and there and finally come to district hospital. There are many families that do not have a means of transport of their own. But now due to 108 [ambulance service] they come. But 108 too come late sometimes. Families say that the vehicle came late or sometimes they tell that it took a long time to arrange for a vehicle.”—a pediatrician lamented [35]. A quote by a block medical officer in a study[35] supports this barrier. He reported that among the tribal population, it is believed that “colostrum causes illness in the newborn by entering the brain of the child,” which acted as a strong deterrent to giving it to the newborn baby. “In institutional deliveries we can do it but in home deliveries we are not able to ensure that. Even in hospitals we have to insist a lot on giving colostrum. They do not give it. They discard it.”
Staffing issues [12,14,18,31,35, 38,39,40,42]	Reflects systemic problems related to the workforce in healthcare, such as inadequate staff numbers, training, or burnout. It indicates that addressing human resource challenges is vital for healthcare delivery. In one of the studies [14], a patient in a focus group discussion said, “Sometimes if one staff takes leave, there won’t be another person to replace her, so one person will have to manage both pharmacy and observation room. It is difficult.” In a study [35], a medical health officer of Satna district (MP) said, “Patients are more and manpower is less. We are working at 40% manpower whether it is doctor or staff nurse. So our care is little affected. We have 50 patients and 2 staff nurses, then care delivery is a little weak. We have expectation from the system because as per the WHO, the ratio of beds to doctors should be there. If this is fulfilled then we can do good work.” A nurse staff in a study [38] quoted, “There are 2 nursing staff for a shift. But the work load is too much. Managing women and infants along with documentation work is really hectic.” In another study [42] done in Odissa, a physician commented that, “without proper staff it’s difficult to manage a UPHC... Now we are 3 staffs here… I, doctor, one pharmacist and one sweeper…. we don’t have any attendant or ANM or staff nurse. If both of them are missing in the same day I face a lot of problems managing alone.”
Case studies of referral management [12,38]	Notable issues in patient transfers between care levels highlight the necessity for improved coordination and communication within the healthcare system. Lack of coordination between lower and higher care levels was evident, with patients often referred without lower-level management. Inefficient referral mechanisms contributed to excessive patient loads at higher care levels. One of the doctors in the study [38] quoted “Women came without any management even at odd hours. Several women came in serious conditions after handled by traditional birth attendants. Further, many women did not have any antenatal care report. Many-a-times they were accompanied by one or maximum two aged females. So making arrangements for medicine, blood and laboratory tests, etc. became difficult. Since the CHWs knew the practice, it was rather easy and fast when they accompany the women.” “Many patients come here for drugs only; there should be a referral mechanism so that they get drugs from other centers. Only those patients who require specialised diabetic care or follow-up for complications should come to a tertiary care facility.”—physician, male, 42 years old [12].
Expressions of staff dissatisfaction [17,23,34,38,39]	This code emphasizes workplace challenges contributing to demotivation and turnover among healthcare workers. It underscores the importance of better working conditions and job satisfaction to uphold service quality, including addressing privacy violations such as inadequate examination spaces and the presence of male providers in maternity wards [38]. A community health worker [38] quoted, “People from village do not agree to male doctor examining female patient. The male gynaecologist needs to intervene only during an emergency…”
Instances of blood bank failures [38]	This highlights critical deficiencies in blood supply management, with potentially life-threatening consequences. It stresses the necessity of robust systems for blood collection, testing, storage, and distribution. A qualitative study [38] in Uttar Pradesh's public health facilities identified poor referral case management, staff shortages, blood bank nonfunctionality, and lack of incentives as barriers to accessing timely healthcare.

The keywords identified from codes related to barriers in accessing healthcare are depicted in the word cloud. The main words getting repeated are "lack," "cost," "knowledge," etc. (Appendices).

Metasynthesis: Network Analysis

**Figure 2:**
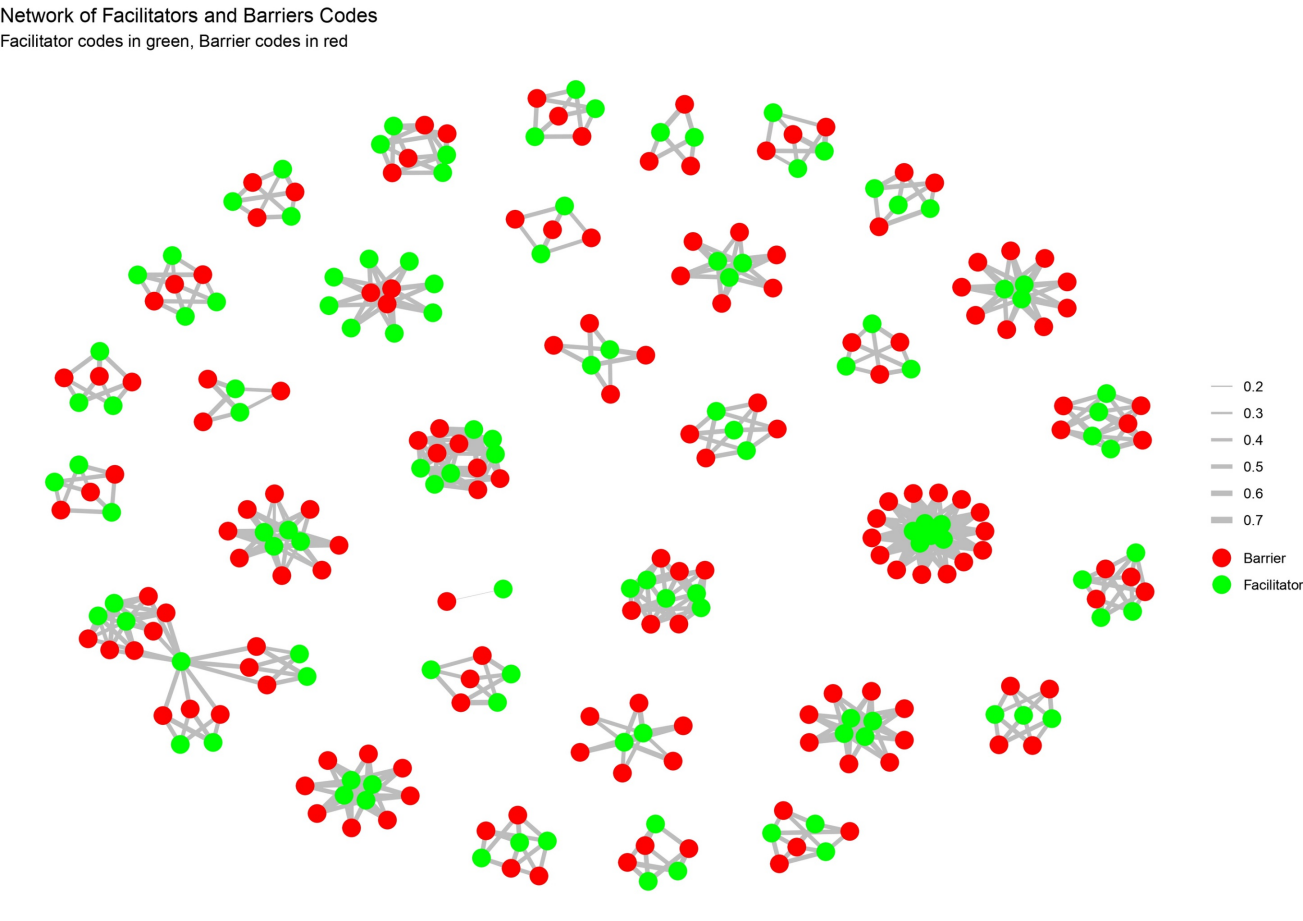
Network diagram showing the interaction between facilitators and barriers (themes and subthemes)

Facilitators such as improved awareness of health entitlements were closely linked to barriers such as confusion around eligibility or operational hurdles. These overlaps highlight a critical point: progress in one area, such as spreading information, may fall short if broader systemic challenges, like administrative efficiency or policy clarity, are not addressed at the same time (Figure 2).

Metasynthesis: Sankey Plot

**Figure 3:**
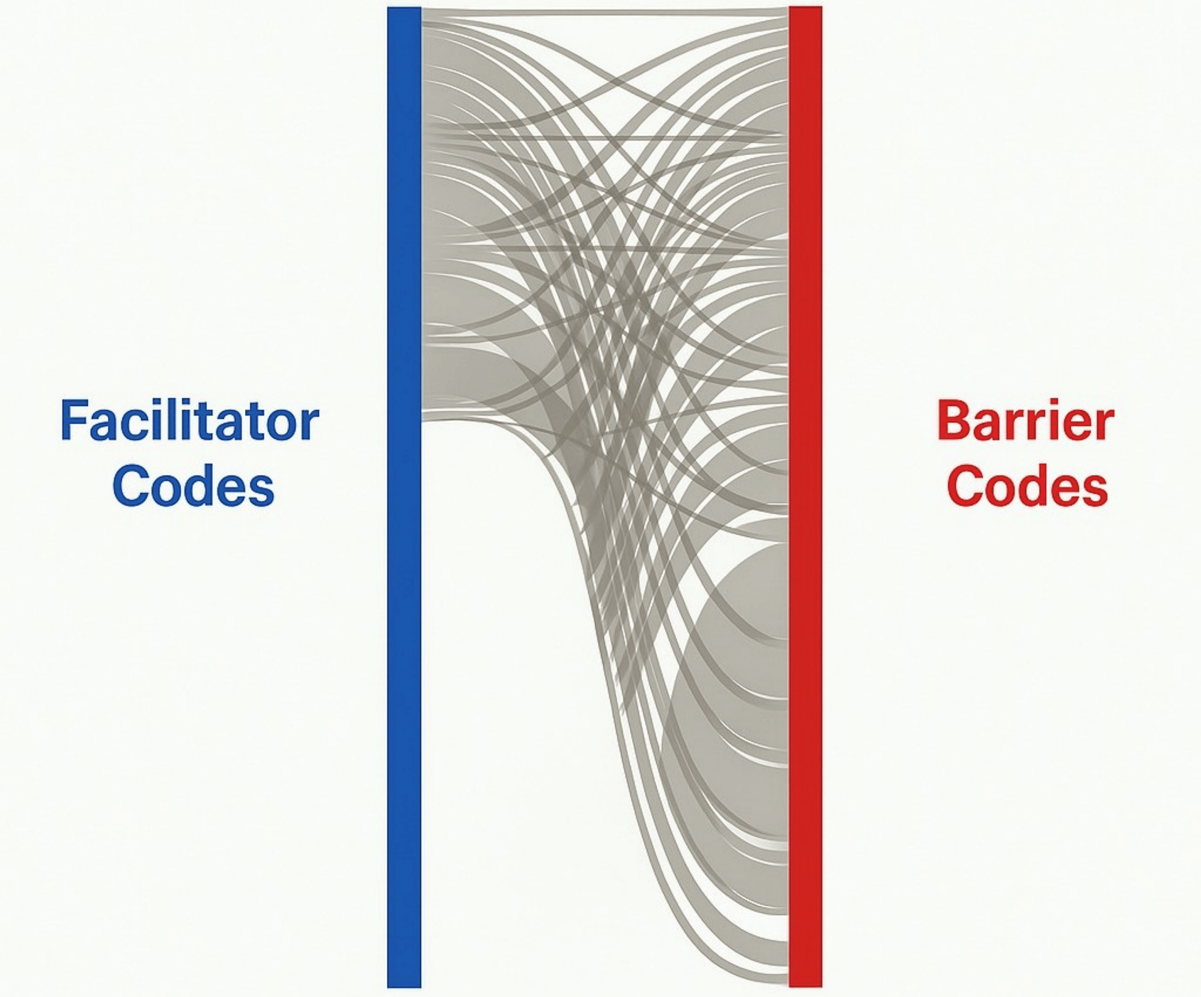
Sankey plot showing interaction between facilitators and barrier themes

The left column of the diagram represents prominent facilitator themes such as accessibility of care, health system support, community engagement, professional development, and availability of resources. These were derived from consistent patterns noted across the dataset, wherein respondents emphasized the presence of infrastructure, supportive staff, and localized outreach efforts as critical enablers of access. The right column depicts major barrier themes, including financial constraints, cultural norms, mistrust in providers, systemic inadequacies, and logistical gaps - barriers that emerged recurrently within interviews, focus groups, and ethnographic observations reported in the included studies (Figure 3).

Discussion

This systematic review delves into healthcare access and utilization in India, identifying critical facilitators such as staff-patient interactions, information sharing, cleanliness, and accessibility. Positive interactions and informed decision-making are pivotal for improving healthcare utilization, necessitating targeted policies to enhance these aspects comprehensively. This review also identified geographically accessibility, difficult transportation options, and a lack of communication and staff training as barriers to healthcare access and utilization in India.

Different lower-middle-income countries face similar challenges in healthcare utilization, where the infrastructure development and presence of trained human resources for health act as key hindrances to healthcare service accessibility and utilization [47,48]. The progress of different countries toward achieving "Health for All" has ensured a certain progress in overcoming those barriers [49].

The interventions are already working by enhancing communication skills training for healthcare providers to ensure clear and patient-centered information sharing. There is also a requirement for community engagement by means of strengthening community outreach programs and collaborating with local leaders to improve healthcare awareness and navigation assistance. It should be coupled with financial accessibility in the form of expanding health insurance coverage and subsidy programs, especially for low-income populations [50,51].

The review proposes several recommendations based on network analysis, such as health literacy investment by implementing health literacy initiatives, particularly community-specific ones, to empower patients, promote healthcare system engagement, and dispel misconceptions. There should be an analysis of patient barriers to suggest improvements in referral processes, infrastructure, or targeted healthcare provider training. There should be integrated interventions addressing the complexity of healthcare access through holistic solutions, considering both staff shortages and patient mistrust due to inadequate health education.

Acknowledging the limitations of qualitative studies, future research employing mixed-methods approaches and diverse geographical coverage is recommended to provide a comprehensive understanding of access disparities. Additionally, evaluating the impact of recent government healthcare initiatives, such as Ayushman Bharat, through similar techniques could offer valuable insights into policy effectiveness.

## Conclusions

This review navigates the multifaceted landscape of healthcare access in India, emphasizing the need for comprehensive strategies to address identified themes. This review identified critical facilitators such as staff-patient interactions, information sharing, cleanliness, and accessibility. This identified geographically accessibility, difficult transportation options, and a lack of communication and staff training as barriers to healthcare access and utilization in India.

## Figures and Tables

**Figure 4:**
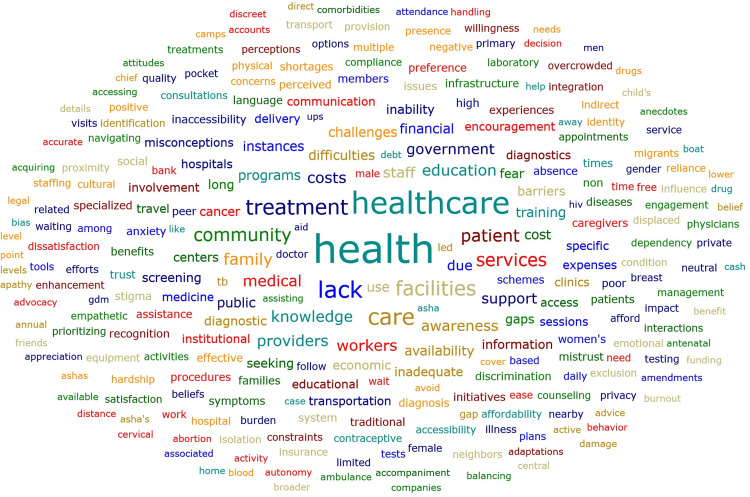
Word cloud generated from codes related to barriers of accessing healthcare facilities

**Figure 5:**
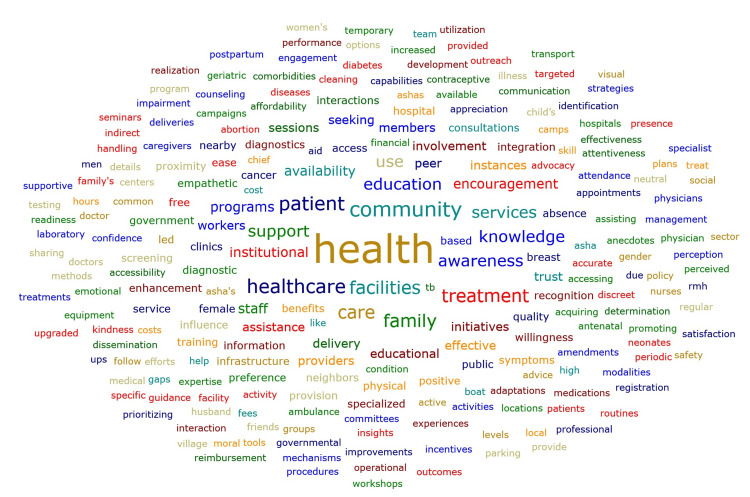
Word cloud generated from codes related to facilitators of accessing healthcare facilities

## Appendices

**Table 7:** Search strategies for the individual databases * Only the first 1000 articles were selected.

Name of database	Search strategy	Year filter	No of articles
PubMed	((("Facilitators"[Title/Abstract] OR "support factors"[Title/Abstract] OR "positive factors"[Title/Abstract] OR "Enablers" [Title/Abstract] OR "Barriers"[Title/Abstract] OR "Hinderances"[Title/Abstract] OR "Challenges"[Title/Abstract]) AND ("Access"[Title/Abstract] OR "Accessing"[Title/Abstract] OR "Using"[Title/Abstract] OR "Utilising"[Title/Abstract] OR "Utilisation"[Title/Abstract])) AND ("public health facilities"[Title/Abstract] OR "health facilities"[Title/Abstract] OR "Healthcare"[Title/Abstract] OR "healthcare facilities"[Title/Abstract] OR "primary health facilities"[Title/Abstract] OR "primary healthcare facilities"[Title/Abstract])) AND (India [Title/Abstract])	2013-2023	634
Scopus	(TITLE-ABS-KEY ( FACILITATORS ) OR TITLE-ABS-KEY ( SUPPORT AND FACTORS ) OR TITLE-ABS-KEY ( POSITIVE AND FACTORS ) OR TITLE-ABS-KEY ( ENABLERS ) OR TITLE-ABS-KEY ( BARRIERS ) OR TITLE-ABS-KEY ( HINDERANCES ) OR TITLE-ABS-KEY ( CHALLENGES ) ) AND ( TITLE-ABS-KEY ( ACCESS ) OR TITLE-ABS-KEY ( ACCESSING ) OR TITLE-ABS-KEY ( USING ) OR TITLE-ABS-KEY ( UTILIZING ) OR TITLE-ABS-KEY ( UTILIZATION ) ) AND ( TITLE-ABS-KEY ( public AND health AND facilities ) OR TITLE-ABS-KEY ( health AND facilities ) OR TITLE-ABS-KEY ( health AND care ) OR TITLE-ABS-KEY ( healthcare AND facilities ) OR TITLE-ABS-KEY ( primary AND health AND facilities ) OR TITLE-ABS-KEY ( primary AND healthcare AND facilities ) ) AND TITLE-ABS-KEY ( india )	2013-2023	1730
Embase	(facilitators:ti,ab,kw OR 'support factors':ti,ab,kw OR 'positive factors':ti,ab,kw OR enabler:ti,ab,kw OR barrier:ti,ab,kw OR hinderance:ti,ab,kw OR challenge:ti,ab,kw) AND (access:ti,ab,kw OR accessing:ti,ab,kw OR using:ti,ab,kw OR utilizing:ti,ab,kw OR utilization:ti,ab,kw) AND ('public health facilities':ti,ab,kw OR 'health facilities':ti,ab,kw OR 'health care':ti,ab,kw OR 'healthcare facilities':ti,ab,kw OR utilization:ti,ab,kw OR 'primary health facilities':ti,ab,kw OR 'primary healthcare facilities':ti,ab,kw) OR (india:ti,ab,kw)	2013-2023	407
Google Scholar^*^	(Facilitators OR “support factors” OR “positive factors” OR Enablers OR Barriers OR Hinderances OR Challenges) AND (Access OR Accessing OR Using OR Utilising OR Utilisation) AND (public health facilities OR health facilities OR Healthcare OR healthcare facilities OR primary health facilities OR primary healthcare facilities) AND (India)	2013-2023	18,700

**Table 8:** Quality assessment of studies using Critical Appraisal Skills Programme (CASP) checklist for qualitative studies Y = Yes.

S. No.	Authors	Year of publication	C1	C2	C3	C4	C5	C6	C7	C8	C9	C10
1	Bhattacharyya et al. [38]	2015	Y	Y	Y	Y	Y	Y	Y	Y	Y	Y
2	Varghese et al. [46]	2015	Y	Y	Y	Y	Y	Y	Y	Y	Y	Y
3	Gawde et al. [44]	2016	Y	Y	Y	Y	Y	Y	Y	Y	Y	Y
4	Vidler et al. [45]	2016	Y	Y	Y	Y	Y	Y	Y	Y	Y	Y
5	Merugumala et al. [21]	2017	Y	Y	Y	Y	Y	Y	Y	Y	Y	Y
6	Nielsen et al. [22]	2017	Y	Y	Y	Y	Y	Y	Y	Y	Y	Y
7	Vellakkal et al. [43]	2017	Y	Y	Y	Y	Y	Y	Y	Y	Y	Y
8	Rath et al. [27]	2018	Y	Y	Y	Y	Y	Y	Y	Y	Y	Y
9	Patel et al. [31]	2018	Y	Y	Y	Y	Y	Y	Y	Y	Y	Y
10	Siddaiah et al. [36]	2018	Y	Y	Y	Y	Y	Y	Y	Y	Y	Y
11	Elias et al. [40]	2018	Y	Y	Y	Y	Y	Y	Y	Y	Y	Y
12	Tripathy et al. [12]	2019	Y	Y	Y	Y	Y	Y	Y	Y	Y	Y
13	Faruqui et al. [20]	2019	Y	Y	Y	Y	Y	Y	Y	Y	Y	Y
14	Jayakumar al., [25]	2019	Y	Y	Y	Y	Y	Y	Y	Y	Y	Y
15	Kung et al. [32]	2019	Y	Y	Y	Y	Y	Y	Y	Y	Y	Y
16	Holloway et al. [11]	2020	Y	Y	Y	Y	Y	Y	Y	Y	Y	Y
17	George et al. [13]	2020	Y	Y	Y	Y	Y	Y	Y	Y	Y	Y
18	Raizada et al. [15]	2020	Y	Y	Y	Y	Y	Y	Y	Y	Y	Y
19	Kathrikolly et al. [17]	2020	Y	Y	Y	Y	Y	Y	Y	Y	Y	Y
20	Boro et al. [18]	2020	Y	Y	Y	Y	Y	Y	Y	Y	Y	Y
21	Santalahti et al. [19]	2020	Y	Y	Y	Y	Y	Y	Y	Y	Y	Y
22	Wood et al. [23]	2020	Y	Y	Y	Y	Y	Y	Y	Y	Y	Y
23	Mukerji et al. [30]	2020	Y	Y	Y	Y	Y	Y	Y	Y	Y	Y
24	Faruqui et al. [33]	2020	Y	Y	Y	Y	Y	Y	Y	Y	Y	Y
25	Dsouza et al. [39]	2020	Y	Y	Y	Y	Y	Y	Y	Y	Y	Y
26	Vijayan et al. [14]	2021	Y	Y	Y	Y	Y	Y	Y	Y	Y	Y
27	Kulkarni et al. [16]	2021	Y	Y	Y	Y	Y	Y	Y	Y	Y	Y
28	Pati et al. [42]	2021	Y	Y	Y	Y	Y	Y	Y	Y	Y	Y
29	Adsul et al. [24]	2022	Y	Y	Y	Y	Y	Y	Y	Y	Y	Y
30	Wani et al. [26]	2022	Y	Y	Y	Y	Y	Y	Y	Y	Y	Y
31	Thiagesan et al. [29]	2022	Y	Y	Y	Y	Y	Y	Y	Y	Y	Y
32	Mohanraj et al. [35]	2022	Y	Y	Y	Y	Y	Y	Y	Y	Y	Y
33	Rajbangshi et al. [37]	2022	Y	Y	Y	Y	Y	Y	Y	Y	Y	Y
34	Cáceres et al. [28]	2023	Y	Y	Y	Y	Y	Y	Y	Y	Y	Y
35	Bangar et al. [34]	2023	Y	Y	Y	Y	Y	Y	Y	Y	Y	Y
36	Saha et al. [41]	2023	Y	Y	Y	Y	Y	Y	Y	Y	Y	Y
